# Retraction: Foliar application of seed water extract of *Nigella sativa* improved maize growth in cadmium-contaminated soil

**DOI:** 10.1371/journal.pone.0286778

**Published:** 2023-06-14

**Authors:** The PLOS ONE Editors

The *PLOS ONE* Editors retract this article [1] because it was identified as one of a series of submissions for which we have concerns about authorship, competing interests, and peer review. We regret that the issues were not addressed prior to the article’s publication.

AMES, MH, and SF did not agree with the retraction. MKH, AA, HMAD, MFA, SH, and NM either did not respond directly or could not be reached.
